# Light Sheet Microscopy-Assisted 3D Analysis of SARS-CoV-2 Infection in the Respiratory Tract of the Ferret Model

**DOI:** 10.3390/v13030529

**Published:** 2021-03-23

**Authors:** Luca M. Zaeck, David Scheibner, Julia Sehl, Martin Müller, Donata Hoffmann, Martin Beer, Elsayed M. Abdelwhab, Thomas C. Mettenleiter, Angele Breithaupt, Stefan Finke

**Affiliations:** 1 Institute of Molecular Virology and Cell Biology, Friedrich-Loeffler-Institut, Federal Research Institute for Animal Health, 17493 Greifswald-Insel Riems, Germany; Luca.Zaeck@fli.de (L.M.Z.); David.Scheibner@fli.de (D.S.); Martin.Mueller@fli.de (M.M.); El-SayedMohammed.AbdEl-Whab@fli.de (E.M.A.); 2 Department of Experimental Animal Facilities and Biorisk Management, Friedrich-Loeffler-Institut, Federal Research Institute for Animal Health, 17493 Greifswald-Insel Riems, Germany; Julia.Sehl@fli.de (J.S.); Angele.Breithaupt@fli.de (A.B.); 3 Institute of Diagnostic Virology, Friedrich-Loeffler-Institut, Federal Research Institute for Animal Health, 17493 Greifswald-Insel Riems, Germany; Donata.Hoffmann@fli.de (D.H.); Martin.Beer@fli.de (M.B.); 4 Friedrich-Loeffler-Institut, Federal Research Institute for Animal Health, 17493 Greifswald-Insel Riems, Germany; ThomasC.Mettenleiter@fli.de

**Keywords:** SARS-CoV-2, COVID-19, 3D immunofluorescence, light sheet microscopy, confocal laser scanning microscopy, tissue optical clearing, host–pathogen interactions, respiratory tract infection

## Abstract

The visualization of viral pathogens in infected tissues is an invaluable tool to understand spatial virus distribution, localization, and cell tropism in vivo. Commonly, virus-infected tissues are analyzed using conventional immunohistochemistry in paraffin-embedded thin sections. Here, we demonstrate the utility of volumetric three-dimensional (3D) immunofluorescence imaging using tissue optical clearing and light sheet microscopy to investigate host–pathogen interactions of pandemic SARS-CoV-2 in ferrets at a mesoscopic scale. The superior spatial context of large, intact samples (>150 mm^3^) allowed detailed quantification of interrelated parameters like focus-to-focus distance or SARS-CoV-2-infected area, facilitating an in-depth description of SARS-CoV-2 infection foci. Accordingly, we could confirm a preferential infection of the ferret upper respiratory tract by SARS-CoV-2 and suggest clustering of infection foci in close proximity. Conclusively, we present a proof-of-concept study for investigating critically important respiratory pathogens in their spatial tissue morphology and demonstrate the first specific 3D visualization of SARS-CoV-2 infection.

## 1. Introduction

In December 2019, a novel coronavirus (2019-nCoV) associated with viral pneumonia emerged in Wuhan, Hubei Province, China [1,2,3,4]. The virus was subsequently designated as severe acute respiratory syndrome coronavirus 2 (SARS-CoV-2) [5] and identified to be the causative agent of COVID-19 (Coronavirus disease 2019). Patients most commonly present with fever, cough, fatigue, and dyspnea [6,7,8,9], while about 17–20% of cases remain asymptomatic throughout the infection [10,11]. About one out of five patients develops severe disease [12,13]. The outbreak was declared a “public health emergency of international concern” on 30 January 2020, and a pandemic on 11 March 2020. As of 2 March 2021, 113,472,187 confirmed cases and 2,520,653 confirmed deaths were reported [14].

SARS-CoV-2 is an enveloped virus with a single-stranded RNA genome of positive polarity and has been classified as a member of the *Coronaviridae* family, genus *Betacoronavirus* [5]. Including SARS-CoV-2, there are currently two alpha- and five betacoronaviruses associated with human disease [15]. While most result in only mild respiratory illness, the three zoonotic betacoronaviruses SARS-CoV [16], SARS-CoV-2 [1,2,3,4], and MERS-CoV [17] (Middle East respiratory syndrome coronavirus) can cause severe respiratory disease. Bats presumably serve as natural reservoir for both SARS-CoV [18,19] and MERS-CoV [20,21,22], whereas palm civets [23] and dromedary camels [24] have been identified as the intermediate hosts for animal–human transmission for SARS-CoV and MERS-CoV, respectively. Viruses closely related to SARS-CoV-2 have been found in bats [2] and Malayan pangolins [25,26], but no direct transmission event or intermediate host species have been identified thus far.

Over the course of the current pandemic, tremendous research efforts have been undertaken to study the virus and its disease. Consequently, critical information on the virus, e.g., receptor usage [27], and necessary research tools, including reverse genetics systems [28,29], became rapidly available. Furthermore, a variety of animal studies to investigate susceptibility and suitability as animal models have been conducted in a number of animal species [30]: ferrets [31,32,33], hamsters [34,35,36], cats [33,37], dogs [33], raccoon dogs [38], rabbits [39], transgenic mice [40,41,42], pigs [32,33], cattle [43], monkeys [44,45,46], poultry [32,33,47], and fruit bats [32].

Within the respiratory tract, detection of viral antigen and RNA suggested a preferential replication of SARS-CoV-2 in the upper respiratory tract (URT) of ferrets [31,32,33], whereas viral antigen was detected in both the URT and lower respiratory tract (LRT) of Syrian hamsters [34,35]. In humans and non-human primates (NHPs), viral antigen detection indicates virus replication in both the URT and LRT [44,48,49].

Thus far, almost all approaches to detect and image SARS-CoV-2 infection in tissues have been based on conventional immunohistochemistry (IHC) of paraffin-embedded thin sections. However, by omitting the spatial context, thin tissue sections of only several micrometers in thickness bear the risk of incomplete or inaccurate description, particularly for focal infections. Recent developments in the field of tissue optical clearing (TOC) have facilitated the preservation of large intact tissue structures by turning them optically transparent. This eliminates the need for physical sectioning and allows acquisition of intact three-dimensional (3D) structures using only optical sectioning, e.g., in light sheet fluorescence microscopy (LSFM) [50]. Lately, the opportunities and advantages of TOC for virus research have been demonstrated in several studies [51,52,53,54,55,56,57]. While two approaches to 3D imaging of SARS-CoV-2-infected lung tissue have been described recently [58,59], neither of them is capable of direct visualization of SARS-CoV-2 infection via virus-specific antigen staining.

In our study, we provide a first complete 3D overview of SARS-CoV-2 infection in the ferret model. By staining for the viral nucleocapsid protein (SARS-CoV-2 N), we were able to directly visualize and localize SARS-CoV-2-infected foci within large volumes of the ferret respiratory tract. Direct visualization further allowed detailed description of the foci in their spatial context. To the best of our knowledge, this is the first report of specific 3D reconstruction of SARS-CoV-2 infection as well as the first report of 3D visualization of respiratory virus infection in nasal turbinates using LSFM.

## 2. Materials and Methods

### 2.1. Cells and Viruses

VeroE6 cells (*Cercopithecus aethiops*; CCLV-RIE 0929, Collection of Cell Lines in Veterinary Medicine [CCLV], Friedrich-Loeffler-Institut, Greifswald-Insel Riems, Germany) were maintained in Minimum Essential Medium with Hanks’ and Earle’s salts (1:1) (Sigma-Aldrich, St. Louis, MO, USA, and Gibco, Waltham, MA, USA, respectively) supplemented with 10% fetal bovine serum (Biowest, Nuaillé, France) and non-essential amino acids (PAN-Biotech, Aidenbach, Germany) in a humidified CO_2_ incubator (37 °C, 5% CO_2_).

SARS-CoV-2 isolate 2019_nCoV Muc-IMB-1 (kindly provided by Roman Wölfel, German Armed Forces Institute of Microbiology, Munich, Germany) was propagated on VeroE6 cells. The complete sequence is available through the GISAID (Global Initiative on Sharing All Influenza Data) database under the accession number ID_EPI_ISL_406862 and name “hCoV-19/Germany/BavPat1/2020”.

### 2.2. Antibodies and Reagents

For the detection of SARS-CoV-2 infection, a 1:1 mixture of hybridoma cell culture supernatants of anti-SARS-CoV-1 N mouse monoclonal antibody clones 4E10A3A1 (RRID:AB_2833160) and 4F3C4 (RRID:AB_2833162) [60] at a dilution of 1:5 or a polyclonal rabbit anti-SARS-CoV-1 N antibody (RRID:AB_838838; Novus Biologicals, Centennial, CO, USA) at a dilution of 1:250 were used. The monoclonal antibodies were raised against amino acid (aa) positions 373-382 (4F3C4) and 400-409 (4E10A3A1), whereas the polyclonal serum was raised against aa positions 399-411 of SARS-CoV-1 N. Alexa Fluor™ 488/568/647-conjugated antibodies against mouse IgG and rabbit IgG were used as secondary antibodies (1:500; Invitrogen, Waltham, MA, USA).

A detailed list of reagents used for the immunostaining and optical clearing of SARS-CoV-2-infected tissue samples is provided in Table S1.

### 2.3. Virus Infection and Immunofluorescence Staining of Mammalian Cell Cultures

For immunofluorescence analysis, 5 × 10^5^ VeroE6 cells were seeded on coverslips one day prior to infection with 1 × 10^6^ TCID_50_ of SARS-CoV-2 isolate 2019_nCoV Muc-IMB-1. Infected VeroE6 cells were then fixed 24 h post-infection with 4% paraformaldehyde (PFA) for 20 min. Following permeabilization with 0.5% Triton X-100/PBS for 15 min, cells were blocked with 10% normal donkey serum in 0.1% Tween-20/PBS (PBS-T) for 30 min. Primary antibodies against SARS-CoV N were applied for 1 h at room temperature in 1% normal donkey serum/PBS-T, followed by three washes with PBS and incubation with the secondary antibody for 1 h at room temperature in 1% normal donkey serum/PBS-T. Nuclei were counterstained with Hoechst33342 (Invitrogen) and samples were embedded in ProLong™ Glass AntiFade Mountant (Invitrogen) for analysis by confocal laser-scanning microscopy.

### 2.4. Tissue Samples of SARS-CoV-2-Infected Ferrets

In a previous study on experimental transmission of SARS-CoV-2 among different animal species, ferrets were inoculated intranasally with 10^5^ TCID_50_ of SARS-CoV-2 isolate 2019_nCoV Muc-IMB-1 [32]. Tissues were collected in 10% neutral-buffered formalin and fixed for at least 21 days to ensure complete virus inactivation. In this study, nasal conchae, trachea, and lung tissue samples from an infected ferret (*n* = 1; referred to as “Ferret 1” by Schlottau and colleagues) euthanized on day 4 post-infection were analyzed. The time point was chosen as it was the only time point of the respective trial where SARS-CoV-2 antigen was reliably detectable. Negative organ material originated from a single naïve animal.

The animal experiments conducted by Schlottau and colleagues [32] were assessed and approved by the ethics committee of the State Office of Agriculture, Food Safety, and Fisheries in Mecklenburg–Western Pomerania (LALLF M-V: LVL MV/TSD/7221.3-2-010/18-12). All procedures were carried out in approved biosafety level 3 facilities.

### 2.5. Immunofluorescence Staining of High-Volume Tissue Sections

Large sections of respiratory tissues (≥150 mm^3^) were immunostained according to a modified iDISCO protocol [52,61]. All incubation steps were conducted with slight agitation and, if not indicated otherwise, at room temperature.

To this end, formaldehyde-fixed tissues were washed three times for at least 1 h each in PBS. Nasal conchae were furthermore decalcified for 4–7 days in Formical-2000™ (Statlab, McKinney, TX, USA). Samples were trimmed to the sizes and volumes described above, and bleached overnight in 5% H_2_O_2_ in PBS at 4 °C. For permeabilization, the tissue samples were first incubated twice for 3 h each with 0.2% Triton X-100/PBS at 37 °C and subsequently in 0.2% Triton X-100/20% DMSO/0.3 M glycine/PBS for 2 days at 37 °C. Following a blocking step with 6% normal donkey serum/0.2% Triton X-100/10% DMSO/PBS for 2 days at 37 °C, primary antibodies were diluted in 3% normal donkey serum/5% DMSO in PTwH (0.2% Tween-20 in PBS with 10 µg/mL heparin) and applied for 4 days at 37 °C. Unbound antibody was removed by washing the samples 4–5 times over the course of a day, leaving the final wash on overnight. Secondary antibodies were diluted in 3% normal donkey serum/PTwH and the samples were incubated for another 4 days at 37 °C. Washing was performed as described for the primary antibody.

### 2.6. Ethyl Cinnamate (ECi)-Based Tissue Optical Clearing

Immunostained tissue sections were cleared with an adjusted ECi-based protocol [62]. All incubation steps were conducted with slight agitation.

The samples were dehydrated in a graded ethanol series (30% [*v*/*v*], 50%, 70%, and twice in 100%; each for ≥8 h at 4 °C, diluted in aqua ad iniectabilia, and pH-adjusted to 9). Following a two-hour wash with *n*-hexane at room temperature [63], *n*-hexane was gradually replaced with the clearing agent ECi and samples were incubated until optically transparent.

### 2.7. Light Sheet Microscopy of Optically Clear Tissue Samples

Light sheet micrographs of optically clear and immunostained respiratory tissues from SARS-CoV-2-infected ferrets were acquired with a LaVision BioTec Ultramicroscope II (LaVision BioTec, Bielefeld, Germany). The microscope was equipped with an Olympus MVX-10 zoom body (magnification range: 0.63×–6.3×, total magnification: 1.26×–12.6×; Olympus, Shinjuku, Tokyo, Japan), an Olympus MVPLAPO 2× objective (NA = 0.5), a LaVision laser module with four laser lines (488 nm, 561 nm, 639 nm, and 785 nm), and a Andor Zyla 5.5 sCMOS Camera (Andor Technology, Belfast, Northern Ireland) with a pixel size of 6.5 µm^2^. To visualize tissue morphology, non-specific autofluorescence was excited with the 488 nm laser. Excitation lines 561 nm and 639 nm were used to excite Alexa Fluor™ 568 and Alexa Fluor™ 647, respectively. Channels of a high-volume 3D image were acquired sequentially with a z-step size of 2 µm, a light sheet width of 100%, and a light sheet thickness of 3.89 µm (NA = 0.156). Acquisition was done with ImSpector (v7.0.124.0).

### 2.8. Confocal Laser-Scanning Microscopy (CLSM)

Confocal images were acquired with a Leica DMI6000 TCS SP5 confocal laser-scanning microscope (Leica Microsystems, Wetzlar, Germany) equipped with a 63×/1.40 oil immersion HCX PL APO objective and a 40×/1.10 water immersion HC PL APO objective. Fluorescence was recorded sequentially between lines with a pinhole diameter of 1 Airy unit and z-step sizes of 0.35 µm. Acquisition was done with LAS AF (v2.7.3.9723).

For high-resolution confocal laser-scanning analysis of cleared and immunostained tissue samples, they were sectioned into 1 mm thick slices using a stainless steel tissue matrix (World Precision Instruments, Hitchin, UK). Tissue slices were then mounted in 3D-printed imaging containers as described before [52]. Tissue morphology was reconstructed from non-specific tissue autofluorescence via excitation with a 405 nm UV laser diode.

### 2.9. Image Processing and Analysis

Image visualization and analysis were performed with arivis Vision4D (v3.2). If necessary, channels were background corrected. CLSM-acquired image stacks of subsectioned volumetric tissue samples were denoised. To quantify relations between SARS-CoV-2 infection foci, they were segmented. The shortest distances between foci were measured using the segment operation “Distances”. To calculate the area of SARS-CoV-2-infected tissue, surface areas of the segmented objects were extracted and divided by two to account only for the surface of the object facing outwards. Lookup tables of multicolor images were selected for maximum accessibility.

## 3. Results

### 3.1. LSFM Provides a Unique Insight into the Spatial Distribution of SARS-CoV-2 in Intact Nasal Turbinates

**Figure 1:**
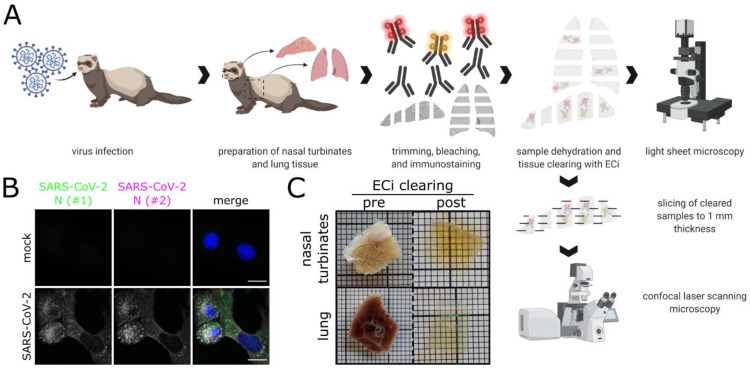
Workflow for correlative LSFM–CLSM of SARS-CoV-2-infected ferret tissues. (**A**) For this study, nasal conchae and lung tissue from SARS-CoV-2-infected ferrets were collected at 4 days post-infection, trimmed, and immunostained against SARS-CoV-2 N protein. Fully dehydrated and optically transparent samples were acquired in toto with a light sheet microscope and subsequently subsectioned to 1 mm thick sections for correlative confocal laser-scanning microscopy. (**B**) Representative immunostaining for SARS-CoV-2 N in infected VeroE6 cells using a commercially available polyclonal anti-SARS-CoV N serum (#1, green) and a monoclonal anti-SARS-CoV N mix (#2, magenta) confirms antibody specificity. Blue: Hoechst33342. Scale bars = 15 µm. (**C**) Representative ferret respiratory tract samples before (left) and after (right) immunostaining and ECi-based optical clearing. The photographs from the lung sections (bottom) show two independent samples. Edge length of grid square: 1 mm.

By combining LSFM with optically cleared samples of the ferret respiratory tract (Figure 1A), we aimed to shed light on the infection environment and spatial context of SARS-CoV-2 infection. A commercially available polyclonal serum (designated as #1), which has been used for SARS-CoV-2 detection by conventional IHC [32,44], and a mix of two monoclonal antibodies (designated as #2) against SARS-CoV N were tested on virus-infected VeroE6 cells and confirmed to be cross-reactive with SARS-CoV-2 N (Figure 1B). Following immunostaining, ferret tissue samples, including the partly ossified nasal conchae, were successfully turned optically transparent using a recently established ethyl cinnamate (ECi)-based approach [62] (Figure 1C).

**Figure 2:**
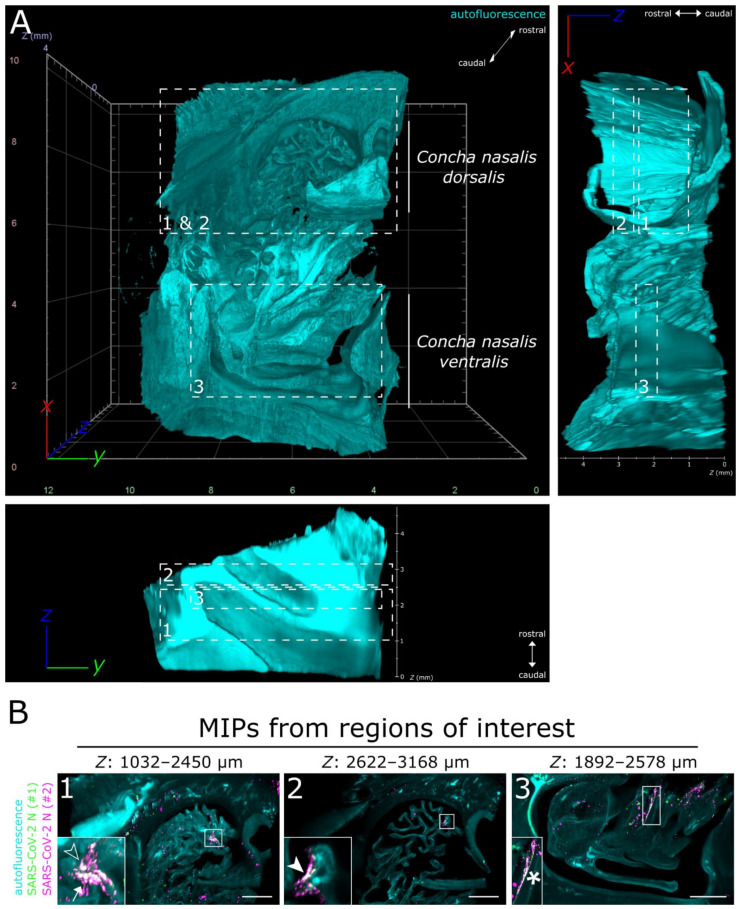
LSFM is able to visualize SARS-CoV-2 infection in nasal turbinates within a high spatial context. (**A**) The tissue structure of the nasal conchae (>200 mm^3^; 4 days post-infection) was reconstructed using tissue autofluorescence (cyan) and is depicted as volumetric projection from three viewing angles. Anatomical terms of location are provided for orientation. Edge length of grid squares = 2 mm. Total magnification = 1.26×. (**B**) Maximum intensity projections (MIPs) of the regions of interest (1–3) highlighted in (A). SARS-CoV-2 infection is characterized by colocalization of both SARS-CoV-2 N stainings (#1, green; #2, magenta) and results in white coloring (inset). Four distinct SARS-CoV-2 infection foci are highlighted (filled-in arrowhead [A1], outlined arrowhead [A5], arrow [A7], and asterisk). Foci will hereafter be referred to via their respective indicator or designation in square brackets. Ranges of the MIPs in the *z*-dimension are provided above the respective image. Scale bar = 1 mm.

Full translucency of ferret nasal turbinates enabled LSFM acquisition of a >200 mm^3^ (6.69 gigavoxels per channel; ∑ = 20.07 gigavoxels)-sized tissue sample (Figure 2A and Movie S1). While there were some unspecific signals detectable in the SARS-CoV-2 N-stained sample (individual green or magenta spots), they could be clearly distinguished from specific SARS-CoV-2 detection by the absence of colocalization (white) of the signals from either antibody (Figure 2B and Figure S1). Within the about 4 mm thick URT sample (4 days post-infection), multiple comparatively small SARS-CoV-2 infection hot spots were visualized (Figure 2B). They were detected in both the *Concha nasalis dorsalis* (Figure 2B, ROIs [region of interests] 1 and 2) and the *Concha nasalis ventralis* (Figure 2B, ROI 3). Overall, these data provide the proof of concept for the feasibility of TOC-assisted LSFM analysis of SARS-CoV-2.

### 3.2. SARS-CoV-2 Infection in the Upper Respiratory Tract of the Ferret Model Is Characterized by an Oligofocal Infection Pattern

**Figure 3:**
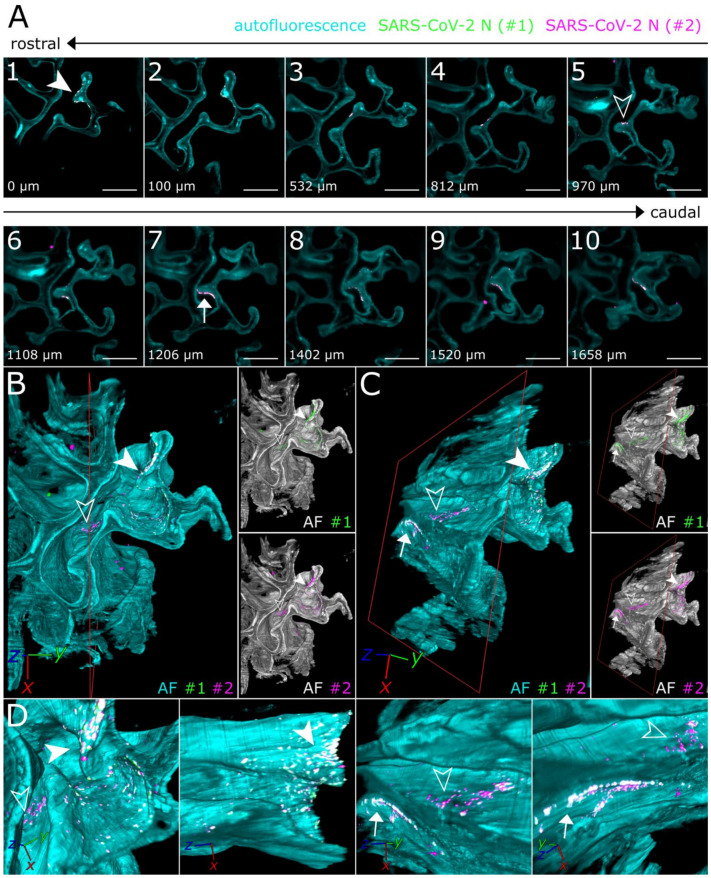
3D detail views highlight oligofocal SARS-CoV-2 infection pattern in nasal turbinates at 4 days post-infection. (**A**) Tomographic representation of three individual SARS-CoV-2 foci (filled-in arrowhead [A1], outlined arrowhead [A5], and arrow [A7]) from ROIs 1 and 2 in Figure 2 along a length of 1658 µm. The relative distance to plane #1 is indicated in the bottom left corner. For additional detail views of the SARS-CoV-2 infection foci A1, A5, and A7, refer to Figure S2. Cyan = autofluorescence; green = SARS-CoV-2 N #1; magenta = SARS-CoV-2 N #2. Scale bar = 400 µm. Total magnification = 8×. (**B**,**C**) Volumetric projections of the detail view from two angles. Clipping at the indicated plane (red) reveals the third SARS-CoV-2 foci (**C**) (arrow), which is hidden behind nasal turbinate tissue in (**B**). Single-channel views further emphasize the colocalizing pattern of both SARS-CoV-2 N stainings (#1, green; #2, magenta). Cyan/grayscale = autofluorescence (AF). (**D**) Close-ups of the three individual infection foci. The angle of the respective image is indicated in the bottom left corner. For single-channel projections, refer to Figure S2.

To achieve a more in-depth analysis of the individual SARS-CoV-2 infection foci, LSFM image stacks of infected areas were acquired using a higher magnification (total magnification of 8× for Figure 3 vs. 1.26× for Figure 2), thus increasing image resolution while maintaining the complete spatial context (Figure 3).

**Figure 4:**
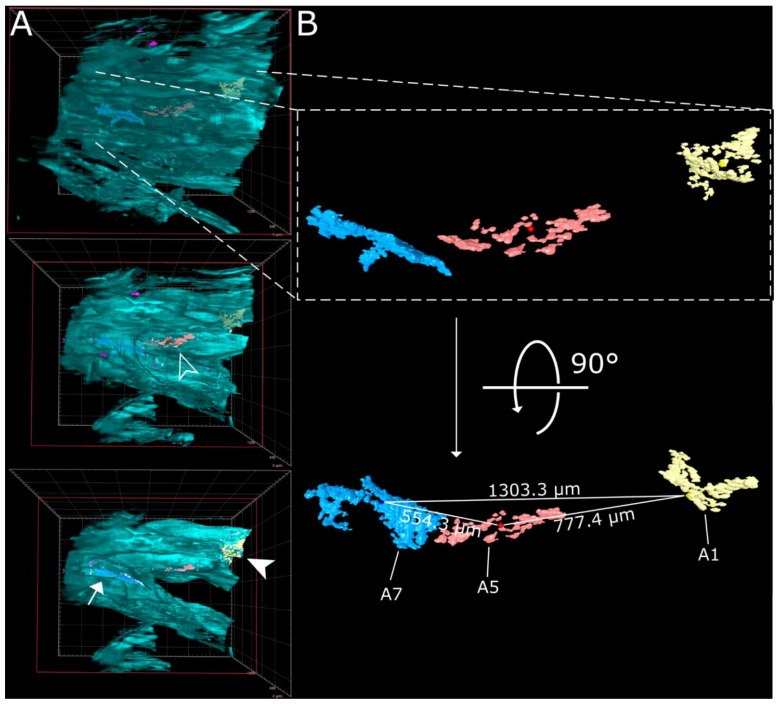
Virtual segmentation of SARS-CoV-2 infection foci at 4 days post-infection. (**A**) xz-view of the magnified nasal turbinate view from Figure 3, clipped at the indicated plane (red square). Segmented SARS-CoV-2 infection foci (A1: yellow, A5: red; A7: light blue) are visible through the autofluorescence reconstruction of the tissue morphology (cyan). Once they are uncovered by the clipping plane, they are highlighted with their respective indicator. (**B**) Detail and alternate viewing angle of segmented infection foci. A slightly darker sphere represents the respective foci center. The direct linear distances between the centers of each foci (from Table 1) are highlighted.

Virtually traveling through an image stack of ROIs 1 and 2 from Figure 2, which was acquired accordingly, corroborated the presence of three individual, well delimitable, and distinguishable SARS-CoV-2 infection foci (Figure 3A, images 1 [filled-in arrowhead], 5 [outlined arrowhead], and 7 [arrow]). A volumetric reconstruction of this image stack was able to convey spatial relationships between the individual infection spots (Figure 3B–D). Specificity of SARS-CoV-2 N detection was again confirmed by colocalization of both independent antibody stainings (Figure 3B,C, right side; for additional detail views and single-channel projections of SARS-CoV-2 infection foci in Figure 3, see Figure S2). Consequently, quantifiable parameters of virtually segmented foci can be assessed (Figure 4). As a proof-of-principle, we quantified the linear distances between foci, and foci areas of SARS-CoV-2 infection spots in the >200 mm^3^-sized nasal turbinate section (Table 1). This increased spatial context within the nasal turbinate sample is further reinforced by the fact that the SARS-CoV-2 infection focus from Figure 3A7 (arrow) is buried deeper within the sample and is not visible from the frontal angle in Figure 3B (Figure 3B,C, red square [clipping plane], and Movie S2). Taken together, this emphasizes the system’s flexibility to switch from broad, mesoscopic overviews to detailed, resolved close-ups. By maintaining the full infection environment, we were able to establish quantifiable relations between the individual SARS-CoV-2 foci, which suggest an oligofocal infection pattern of SARS-CoV-2 in the URT of ferrets.

### 3.3. CLSM Acquisition of Correlated Regions of Interest at Subcellular Resolution–Infection of Ciliated and Non-Ciliated Cells in the Nasal Epithelium

While LSFM is ideally suited to generate a mesoscopic overview to analyze, for example, large-scale spatial virus distribution within virus-infected tissues, simultaneous resolution of subcellular details is not possible. Thus, following LSFM acquisition, we subsectioned the optically cleared high-volume tissue sample to 1 mm thick slices using a tissue matrix to achieve compatibility with the limited free working distances of CLSM objectives (Figure 1A).

**Figure 5:**
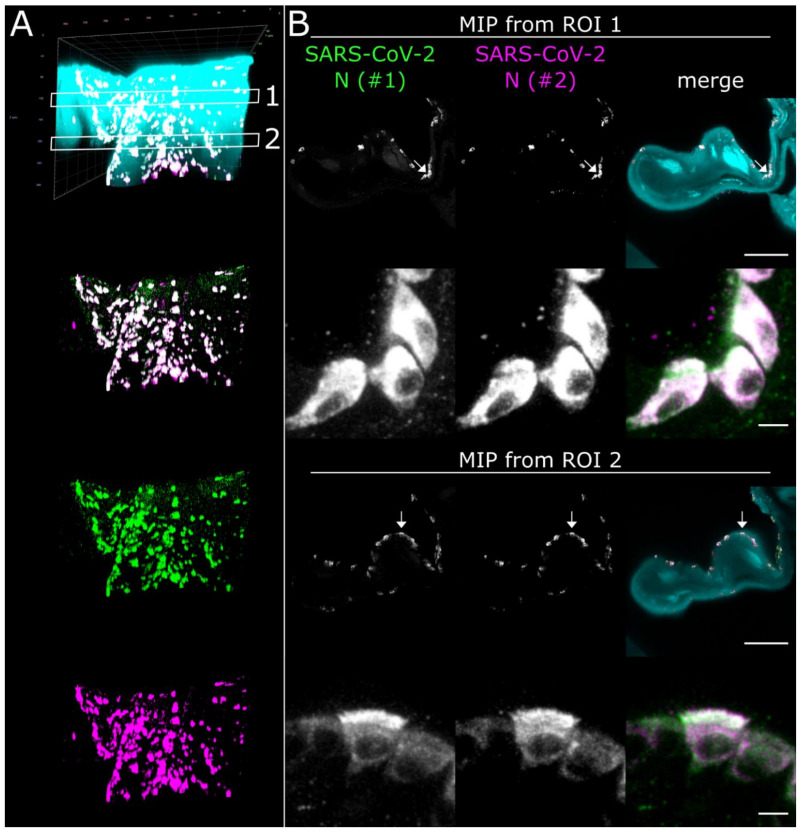
High-resolution CLSM analysis of SARS-CoV-2 infection foci in the *Concha nasalis dorsalis* of a SARS-CoV-2-infected ferret at 4 days post-infection. (**A**) 3D maximum intensity projection (MIP) of a SARS-CoV-2 infection focus from ROI 1 in Figure 2. The image stack was acquired with a 40×/1.1 water immersion objective. Cyan = autofluorescence; green = SARS-CoV-2 N #1; magenta = SARS-CoV-2 N #2. Edge length of grid square = 40 µm. (**B**) MIPs from ROIs 1 and 2 in (**A**). Individual cells can be analyzed at subcellular resolution, highlighting infection of ciliated and non-ciliated cells (arrows). For z-stack analysis of ROI 2, refer to Figure S3. Scale bar = 100 µm (overview) and 5 µm (detail).

Using the spatio-morphological information on the distribution of SARS-CoV-2 infection foci obtained from LSFM analysis, high-resolution CLSM image stacks of a SARS-CoV-2 infection focus in the *Concha nasalis dorsalis* (ROI 1 from Figure 2) were acquired (Figure 5). Individual SARS-CoV-2-infected cells could be resolved, demonstrating cytoplasmic SARS-CoV-2 N distribution in both ciliated and non-ciliated cells (Figure 5B, arrows; for additional detail views, see Figure S3 and Movie S3). Notably, SARS-CoV-2 N accumulated particularly at the apical side of the ciliated cells. Overall, these data demonstrate the feasibility of this correlated approach to dissect cell-specific responses to SARS-CoV-2 infection in vivo at subcellular resolution.

### 3.4. SARS-CoV-2 Detection in the Lower Respiratory Tract of Ferrets

Previous studies demonstrated a preferential replication of SARS-CoV-2 in the URT of ferrets [31,32,33]. To assess whether comprehensive LSFM analysis may uncover previously undetected SARS-CoV-2 infection foci in the LRT, we looked at optically cleared high-volume lung and tracheal samples.

**Figure 6:**
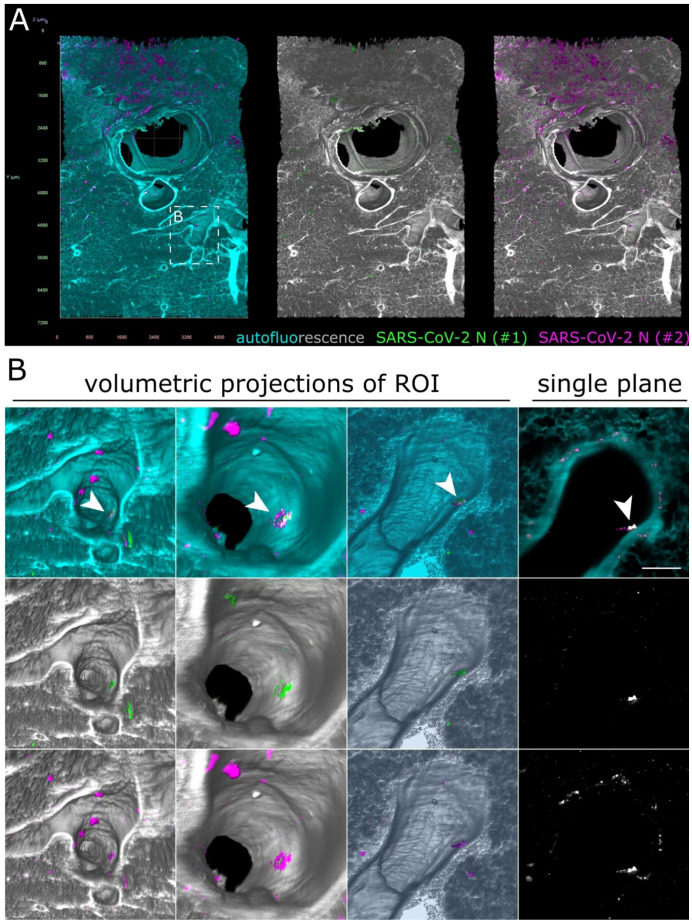
Only debris-associated SARS-CoV-2 antigen was detectable in ferret lung tissue at 4 days post-infection. (**A**) Volumetric projection of a large lung tissue section. While some background staining is detectable for the monoclonal antibody mix (#2, magenta), no signal overlap with the polyclonal antibody (#1, green) is visible. Cyan/grayscale = autofluorescence. Edge length of grid squares = 800 µm. Total magnification = 1.6×. (**B**) Alternate viewing angles reveal a spot inside an airway where both signals colocalize (white box in (**A**)). Contrary to the SARS-CoV-2-associated foci in Figure 2 and Figure 3, the overlapping signal is detected lying on top of the epithelial layer, suggesting that it is most likely cell debris inhaled from the URT.

As before, some unspecific fluorescence signals could be seen in both lung (Figure 6) and tracheal tissue (Figure S4 and Movie S4). At first glance, no specific SARS-CoV-2 infection foci could be identified. However, hidden within an airway of the large lung tissue volume, a 172 µm by 102 µm-sized spot of colocalized antibody signals was detected (Figure 6B and Movie S5). Contrary to the SARS-CoV-2 infection foci in the ferret nasal turbinates (Figure 2 and Figure 3), the signal was localized above the epithelial cell layer (Figure 6B, single plane). This suggested detection of debris-associated antigen, which was most likely inhaled from the URT. Overall, while we were able to detect an 8.6 × 10^−5^ mm^3^ (86,000 µm^3^) spot of debris-associated antigen within a >80 mm^3^ volume, we did not identify additional sites of infection within the LRT of the ferret, which corroborates the previously described preferential replication of SARS-CoV-2 in the URT of ferrets [31,32,33].

## 4. Discussion

While conventional immunohistochemistry studies have been used to assess the presence or absence of SARS-CoV-2 antigen in human and animal tissues [31,32,33,34,35,44,48,49], none of them were able to provide a greater spatial context of the infection site. By combining TOC with LSFM, we acquired large intact volumes of SARS-CoV-2-infected respiratory tissues from the ferret animal model (Figure 1). The direct 3D visualization of virus infection via SARS-CoV-2 N staining established a comprehensive and mesoscopic overview of the infection in its full spatial context (Figure 2, Figure 3, Figure 4, Figure 5 and Figure 6 and Movies S1–S5). Moreover, the determination of morphological parameters, e.g., focus-to-focus distances or the area of virus-infected tissue, not only allowed the characterization of individual SARS-CoV-2 infection foci but also provided a first quantitative insight into virus distribution within the spatio-morphological context of ferret nasal turbinates (Figure 4 and Table 1).

Here, we employed an ECi-based TOC approach [62] and adjusted it to visualize immunostained SARS-CoV-2 infection in large tissue samples of the respiratory tract of ferrets. While two 3D imaging approaches to SARS-CoV-2 infection in lung tissue have been reported, they have an entirely different scope: as both represent virtual histopathology strategies, they are meant to assess pathophysiology and associated tissue damage, but inherently cannot map and visualize specific SARS-CoV-2 infection. For the first study, Eckermann et al. [58] developed and demonstrated the utility of a phase-contrast x-ray tomography concept to investigate unstained lung tissue. The second study describes the use of fluorescent H&E-analog stains (TO-PRO-3 for nuclear contrast and Eosin-Y for cytoplasmic/stromal contrast) to achieve “3D pseudo-histological imaging” [59]. Consequently, by providing the 3D distribution of specific SARS-CoV-2 infection, our study constitutes the first report of direct 3D visualization of SARS-CoV-2 infection via LSFM. While unspecific antibody signals were detected for both the polyclonal serum and the monoclonal mix directed against N of SARS-CoV, particularly on the outer surface of the tissue blocks, specificity for SARS-CoV-2 was ensured via colocalization of two independent antibody stainings (Figure 2, Figure 3, Figure 5 and Figure 6, Figures S2 and S3) and absence of any colocalization in naïve animals (Figure S1). Further optimization of the immunostaining protocol or the availability of SARS-CoV-2-specific antibodies will likely aid in reduction of background staining and improvement of virus detection.

In addition to the specific 3D reconstruction of SARS-CoV-2 infection within its spatio-morphological environment, the implementation of quantitative image analysis following accurate quantification of interrelated 3D parameters (Figure 4 and Table 1) represents a pronounced advantage of 3D immunofluorescence imaging over conventional IHC. To that effect, quantitative 3D measurements like linear distances can serve as a quantifiable marker of proximity in an open-space compartment such as the nasal turbinates. To achieve a somewhat comparable yet more artifact-prone 3D reconstruction from thin sections, exceedingly laborious and time-consuming image registration pipelines following serial thin sectioning are necessary [64]. For instance, the nasal turbinate section from Figure 2, Figure 3 and Figure 4 alone would require processing of around 800 sections (at 5 µm thickness), making it de facto impossible with 2D IHC.

When compared to previous studies [31,32,33], the spatial visualization of SARS-CoV-2 in the ferret respiratory tract confirmed preferential infection of the URT (Figure 2, Figure 3 and Figure 4). Furthermore, our data indicate a distinct oligofocal infection pattern of SARS-CoV-2 within nasal turbinates (Figure 2, Figure 3 and Figure 4). Within a >200 mm^3^ section of nasal turbinate tissue, only four SARS-CoV-2 infection foci (with a combined volume of 5.17 × 10^−3^ mm^3^) were detected, three of which accumulated in the *Concha nasalis dorsalis* and exhibited a maximum linear distance of 1.3 mm to each other (Figure 4 and Table 1). It is important to note that tissues inevitably shrink during the fixation, dehydration, and clearing process. For the EtOH-ECi-based TOC approach used here, a 50% volume reduction, equaling to a change of about 20% in tissue diameters, has been determined [62]. The limited degree of infection is particularly interesting in view of the amounts of infectious virus and genome copies that can be isolated from the URT of ferrets [31,32,33] and other animal species, like Syrian hamsters [34,35,36] and rhesus macaques [44,45,46]. Clustering of SARS-CoV-2 infection foci in narrow areas of the URT might also have implications for the likelihood of isolation of infectious virus and the detection of viral RNA from nasal swabs in comparison to nasal washes from ferrets and possibly other animal models. Accordingly, a high degree of variation in viral copy numbers can be observed from nose or throat swabs in comparison to bronchoalveolar lavages from SARS-CoV-2-infected rhesus macaques [44]. However, because of the proof-of-principle character of our study and the limited availability of SARS-CoV-2-infected material, further studies have to corroborate the clustering and focal infection pattern of SARS-CoV-2 in the URT.

Alongside complex quantitative 3D image analysis, volumetric imaging has the potential to discover rare events, as demonstrated by the detection of cancer metastases in sentinel lymph nodes, which had not been found via conventional IHC [65]. While preferential replication of SARS-CoV-2 in the URT of ferrets has been demonstrated via viral RNA and antigen detection [31,32,33], Kim et al. also detected some SARS-CoV-2-positive cells in the LRT. This is in contrast to the two other ferret susceptibility studies, which detected either no [33] or only low amounts [32] of viral RNA in the LRT, but neither found any SARS-CoV-2 antigen at this location. It is conceivable that scarce LRT infection had been overlooked in previous 2D IHC studies because of the focal character of SARS-CoV-2 infection in the tissue or that the detected viral RNA originated from URT-derived aspirated material. Using this high-volume imaging approach, we aimed to screen the tissue for rare SARS-CoV-2 infection foci in the LRT. While we did not detect any infection spots in tracheal tissue (Figure S4), we did visualize an individual, only 86,000 µm^3^-sized SARS-CoV-2 N-positive structure inside a lung airway (Figure 6B). However, spatial analysis revealed that the signal, contrary to the SARS-CoV-2 infection foci in the nasal turbinate epithelium (Figure 2 and Figure 3), was detected above the airway epithelial layer. This strongly suggested that the structure most likely represents aspirated virus-containing debris as the result of localized cell or tissue damage at infected URT sites. Accordingly, this example emphasizes the suitability of this volumetric 3D LSFM approach to identify rare and highly localized pathogen-related events.

Ferrets are a standard model for human respiratory infection [66]. However, they recapitulate only mild SARS-CoV-2 infection and do not develop severe respiratory disease [13,31,32,33]. In contrast, both URT and LRT are strongly affected by SARS-CoV-2 infection in Syrian hamsters, including overt signs of disease [34,35]. This is closer to human disease, where SARS-CoV-2 antigen is found in the URT and LRT, as corroborated by the NHP model rhesus macaques [44,45,46]. While this proof-of-principle study is focused on ferret samples, it may serve as a blueprint for further analyses in other animal models and even human clinical samples.

Independent of sample origin, volumetric imaging of cleared samples enables discovery and detection of rare infection events, as demonstrated earlier. This facilitates the investigation of the involvement of other organs outside of the respiratory tract in SARS-CoV-2 infection. Recently, extrapulmonary manifestations of COVID-19 became the focus of attention [67]. Accordingly, SARS-CoV-2 antigen has been detected in the central nervous system (CNS) of humans and hACE (human angiotensin-converting enzyme 2)-knockin mice [41,68]. Infection of the CNS might occur via the olfactory nerve as viral antigen has been found in the olfactory mucosa of humans and experimentally infected Syrian hamsters [69,70], including in olfactory sensory neurons [69]. Additionally, viral antigen has been detected in the intestine of ferrets [31], hamsters [34,35], and rhesus macaques [44]. Previous studies with other viral pathogens demonstrated that volumetric 3D imaging using TOC and LSFM is a highly valuable tool to assess the comprehensive distribution of virus infection in vivo [51,54]. Additional immunostaining against tissue-specific cell markers may further facilitate the investigation of the global SARS-CoV-2 cell tropism in affected tissues. Combining and correlating this with high-resolution CLSM analysis of SARS-CoV-2 hot spots identified via LSFM has the potential to dissect subcellular infection processes of SARS-CoV-2 in vivo with unparalleled detail. These results form the basis for research on larger sample sizes of both respiratory and non-respiratory tissues from SARS-CoV-2 animal models and human clinical samples using volumetric 3D LSFM of immunostained and cleared tissues.

Overall, we demonstrate the proof of concept for the utility of volumetric 3D immunofluorescence of critically important respiratory pathogens such as SARS-CoV-2 using TOC and LSFM. The ability to analyze interrelated morphological parameters, like inter-foci distances and SARS-CoV-2-affected areas, and to put them into global perspective to the spatial tissue morphology, provides unprecedented insight into SARS-CoV-2 infection in the respiratory tract of ferrets. In the future, this approach will be a crucial tool to understand the mesoscopic scale of host–pathogen interactions of SARS-CoV-2, but also other respiratory and non-respiratory pathogens, including, for example, influenza A virus and henipaviruses.

## Figures and Tables

**Table 1:** Direct linear distances between, areas affected by, and volumes of segmented SARS-CoV-2 infection foci. Linear distances were calculated either as the distance between the center of two foci or as the shortest possible distance between the edges of two foci. The area affected by SARS-CoV-2 infection was measured by calculating the surface area of segmented objects and dividing the resultant value by two, thus only accounting for the surface facing outwards.

Linear Distance [µm]		A1	A5	A7
from the center	A1	0	777.4	1303.3
	A5	777.4	0	554.3
	A7	1303.3	554.3	0
from the edge	A1	0	421.1	997.5
	A5	421.1	0	75.5
	A7	997.5	75.5	0
**Area [µm^2^]**		90,212	75,389	181,431
**Volume [µm^3^]**		465,818	443,207	1,533,316

## Data Availability

The datasets are available from the corresponding author on reasonable request.

## Supplementary Materials

The following are available online at https://www.mdpi.com/1999-4915/13/3/529/s1, Figure S1: No SARS-CoV-2 colocalization of antibody signals is observable in tissue from mock-infected control animals. Figure S2: Close-up details and single-channel projections of SARS-CoV-2 infection foci. Figure S3: SARS-CoV-2 N was diffusely distributed in the cytoplasm of the infected nasal turbinate epithelium. Figure S4: No SARS-CoV-2 infection foci were detected in ferret tracheal tissue at 4 days post-infection. Table S1: List of reagents and antibodies used for immunostaining and optical clearing of tissue from SARS-CoV-2-infected ferrets. Movie S1: Volumetric 3D projection of an LSFM-acquired, >200 mm^3^-sized nasal turbinate section from a SARS-CoV-2-infected ferret. Movie S2: Fly-through animation of individual SARS-CoV-2 infection foci in ferret nasal turbinates at 4 days post-infection. Movie S3: Tomography view of a CLSM-resolved, SARS-CoV-2-infected cell in the nasal epithelium of a ferret at 4 days post-infection. Movie S4: 360° rotation of a trachea section from a SARS-CoV-2-infected ferret at 4 days post-infection. Movie S5: Fly-through animation of likely debris-associated SARS-CoV-2 infection in ferret lung tissue.
