# Supplementary material for: Light Sheet Microscopy-Assisted 3D Analysis of SARS-CoV-2 Infection in the Respiratory Tract of the Ferret Model
Source: Viruses. 2021 Mar 23;13(3):529. doi: 10.3390/v13030529 (PMC8004956; doi:10.3390/v13030529)
Supplement: Supplementary file 1 [file viruses-13-00529-s001.zip › Supplementary-material.docx]

**Supplementary Material**

**Light Sheet Microscopy-Assisted 3D Analysis of SARS-CoV-2 Infection in the Respiratory Tract of the Ferret Model**

Luca M. Zaeck^1^, David Scheibner^1^, Julia Sehl^2^, Martin Müller^1^, Donata Hoffmann^3^, Martin Beer^3^, Elsayed M. Abdelwhab^1^, Thomas C. Mettenleiter^4^, Angele Breithaupt^2^ and Stefan Finke^1,^*

^1^ Institute of Molecular Virology and Cell Biology, Friedrich-Loeffler-Institut, Federal Research Institute for Animal Health, 17493 Greifswald-Insel Riems, Germany

^2^ Department of Experimental Animal Facilities and Biorisk Management, Friedrich-Loeffler-Institut, Federal Research Institute for Animal Health, 17493 Greifswald-Insel Riems, Germany

^3^ Institute of Diagnostic Virology, Friedrich-Loeffler-Institut, Federal Research Institute for Animal Health, 17493 Greifswald-Insel Riems, Germany

^4^ Friedrich-Loeffler-Institut, Federal Research Institute for Animal Health, 17493 Greifswald-Insel Riems, Germany

* Correspondence: Stefan.Finke@fli.de

**Supplementary Movie Legends**

**Movie S1: Volumetric 3D projection of an LSFM-acquired, > 200 mm^3^-sized nasal turbinate section from a SARS-CoV-2-infected ferret.** The tissue morphology was reconstructed using non-specific tissue autofluorescence (cyan). Edge length of grid squares = 2 mm. Total magnification = 1.26x.

**Movie S2: Fly-through animation of individual SARS-CoV-2 infection foci in ferret nasal turbinates at 4 days post-infection.** The three distinct SARS-CoV-2 infection foci from Figures 3 and 4 are highlighted at timestamps (mm:ss) 00:09 [A1], 00:14 [A5], and 00:25 [A7]. Cyan = autofluorescence; green = SARS-CoV-2 N #1; magenta = SARS-CoV-2 N #2. Edge length of grid squares = 300 µm. Total magnification = 8x.

**Movie S3: Tomography view of a CLSM-resolved, SARS-CoV-2-infected cell in the nasal epithelium of a ferret at 4 days post-infection.** The z-stack analysis of an infected cell from Figure 5B (ROI 2) emphasizes cytoplasmic distribution of SARS-CoV-2 N within the cell. The nucleus is largely excluded. For individual planes, refer to Figure S3. Green = SARS-CoV-2 N #1; magenta = SARS-CoV-2 N #2. Edge length of grid squares = 2 µm

**Movie S4: 360° rotation of a trachea section from a SARS-CoV-2-infected ferret at 4 days post-infection.** No SARS-CoV-2-associated infection spots were detected. Cyan = autofluorescence; green = SARS-CoV-2 N #1; magenta = SARS-CoV-2 N #2. Edge length of grid squares = 800 µm. Total magnification = 2x.

**Movie S5: Fly-through animation of likely debris-associated SARS-CoV-2 infection in ferret lung tissue.** The colocalization of either SARS-CoV-2 N antibody signal (#1, green; #2, magenta) inside a lung airway can be observed at timestamp (mm:ss) 00:16. Cyan = autofluorescence. Edge length of grid squares = 200 µm. Total magnification = 6.4x.


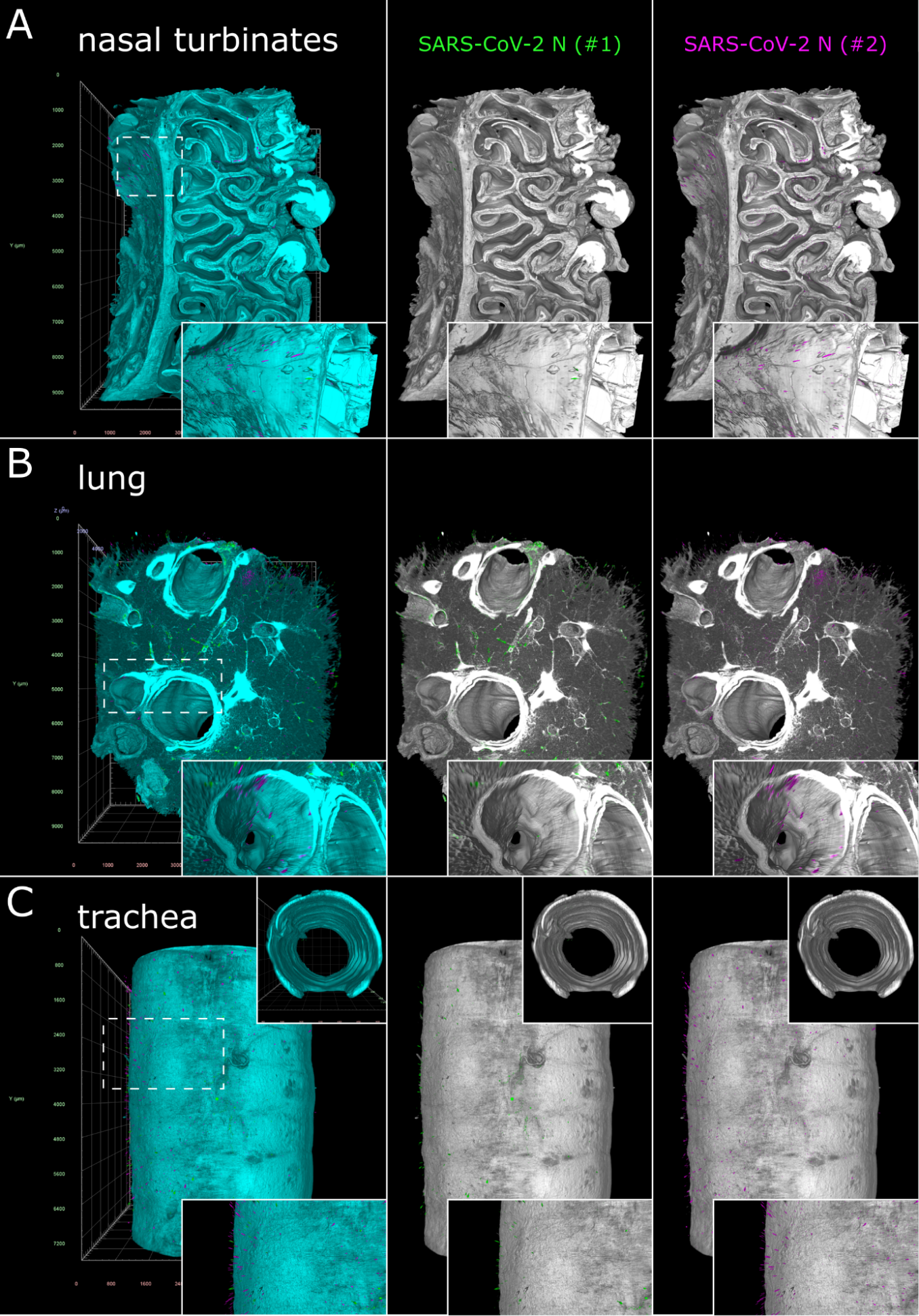


**Figure S1: No SARS-CoV-2 colocalization of antibody signals is observable in tissue from mock-infected control animals.** Volumetric projections of nasal turbinate **(A)**, lung **(B)**, and trachea **(C)** tissue from mock-infected control animals. No colocalization of the polyclonal serum (#1, green) and the monoclonal mix (#2, magenta) was detected. Cyan/grayscale = autofluorescence. Edge length of grid squares = 1,000 µm. Total magnification = 1.6x (A and B), 2x (C).


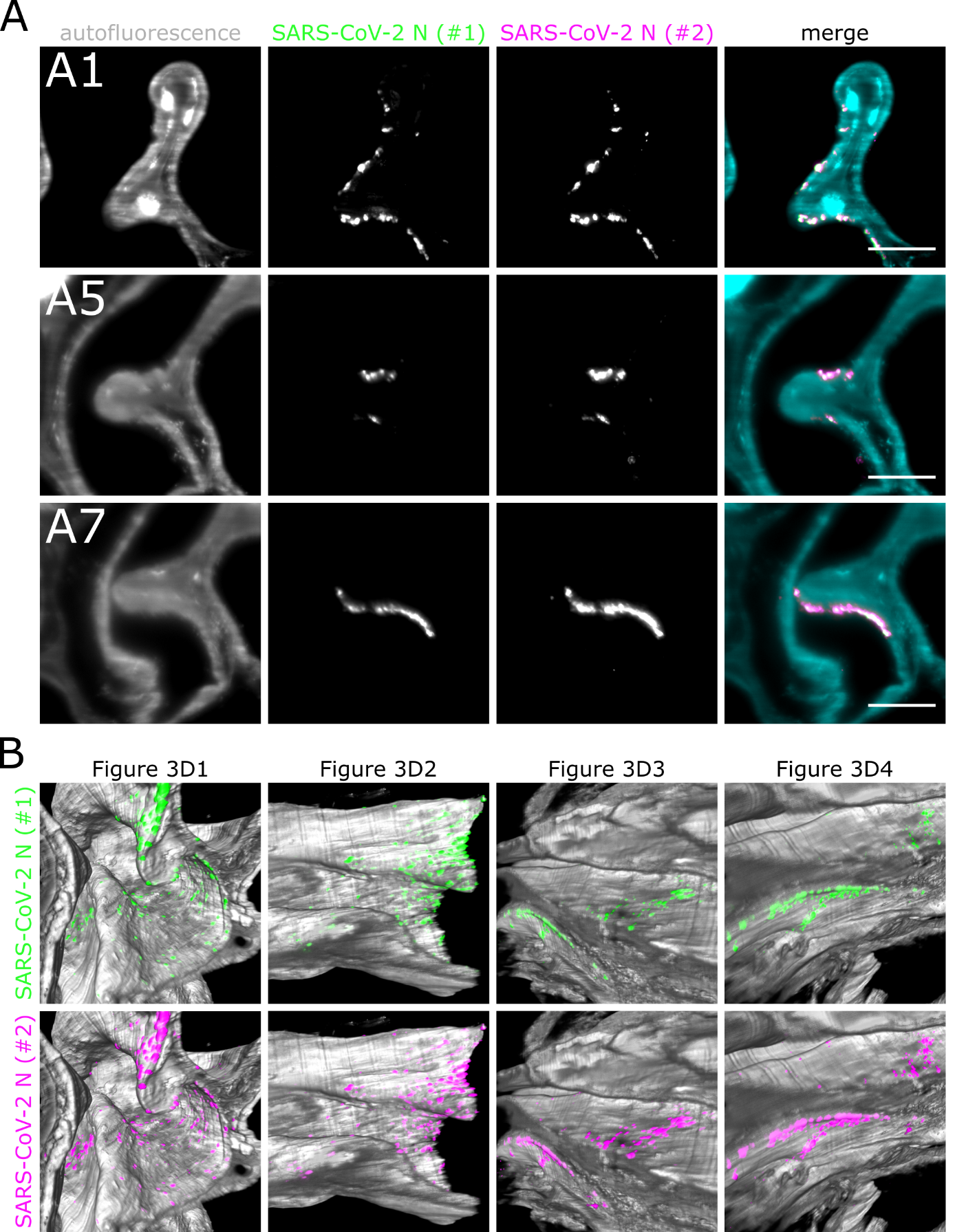


**Figure S2: Close-up details and single-channel projections of SARS-CoV-2 infection foci.** **(A)** Detail views of individual SARS-CoV-2 infection foci in ferret nasal turbinate tissue from Figure 3A (relating to images #1, 5, and 7). The colocalization of both antibody signals confirmed specific detection of SARS-CoV-2 infection. Cyan = autofluorescence; green = SARS-CoV-2 N #1; magenta = SARS-CoV-2 N #2. Scale bar = 150 µm. **(B)** Single-channel views of volumetric projections from Figure 3D. Grayscale = autofluorescence; green = SARS-CoV-2 N #1; magenta = SARS-CoV-2 N #2.


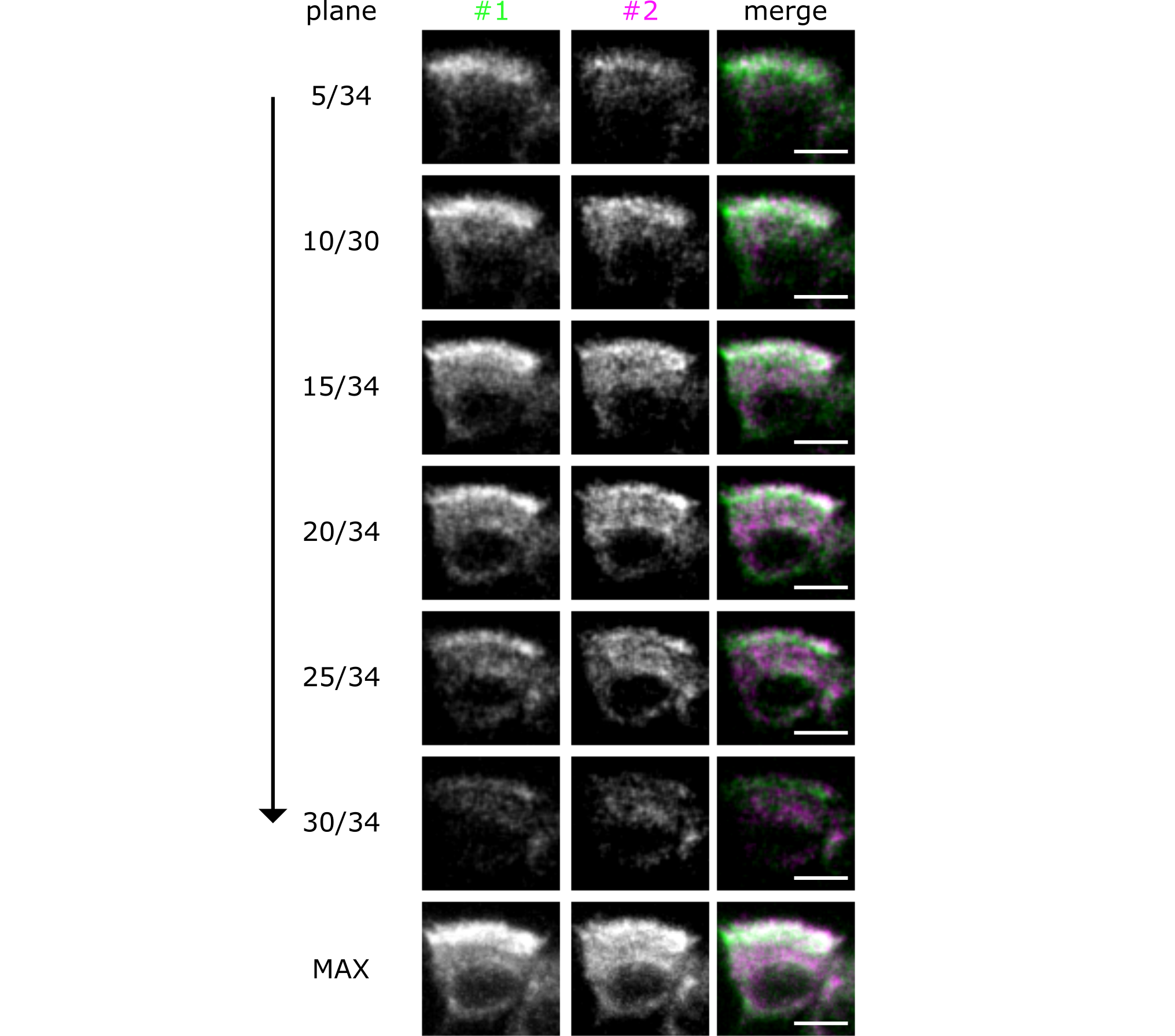


**Figure S3: SARS-CoV-2 N was diffusely distributed in the cytoplasm of the infected nasal turbinate epithelium.** Z-stack analysis of the infected cells from Figure 5B (ROI 2) highlighted the cytoplasmic distribution of SARS-CoV-2 N in infected cells of the nasal epithelium. The nucleus was largely excluded. Cyan = autofluorescence; green = SARS-CoV-2 N #1; magenta = SARS-CoV-2 N #2. Scale bar = 5 µm.


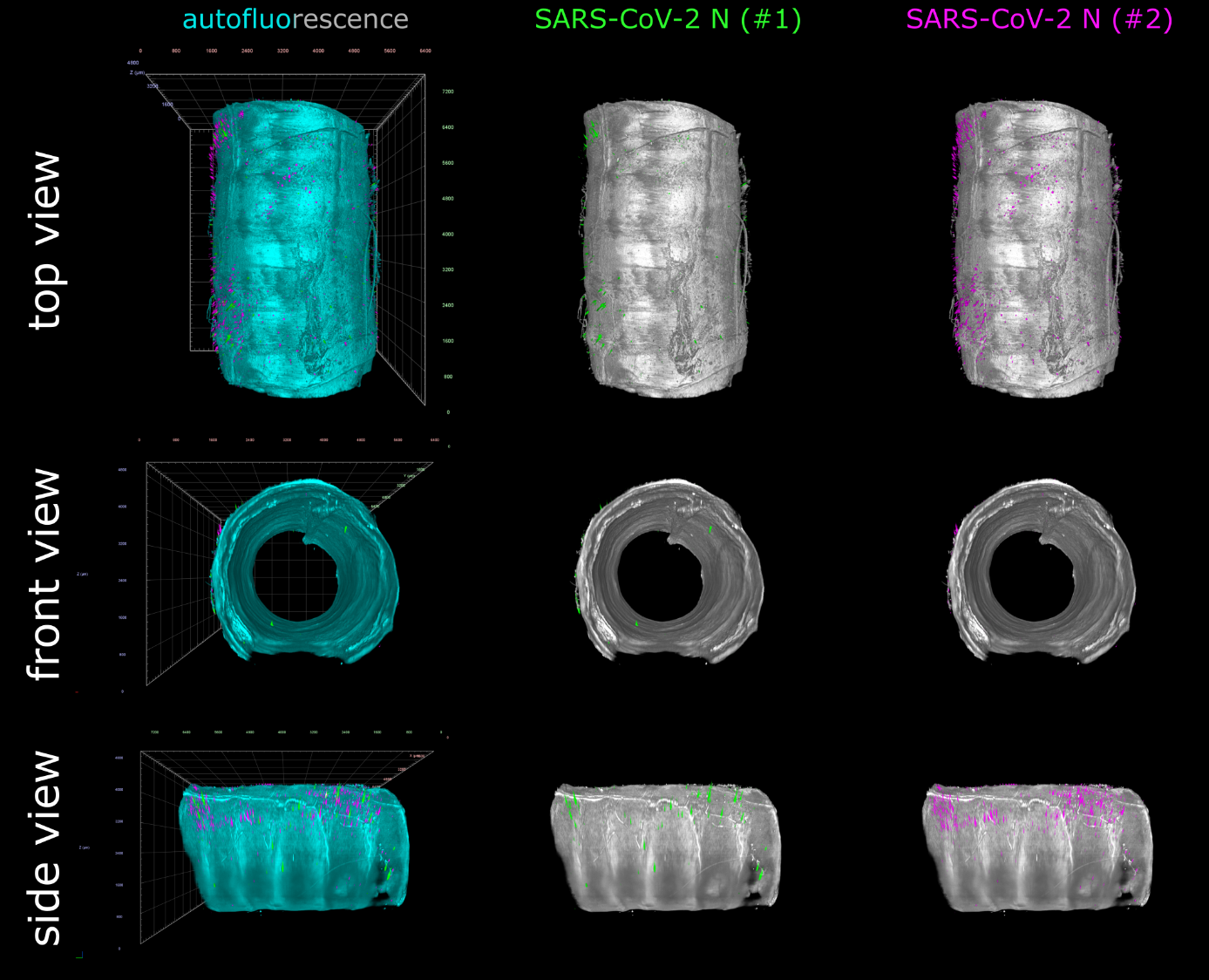


**Figure S4: No SARS-CoV-2 infection foci were detected in ferret tracheal tissue at 4 days post-infection.** Volumetric projection of a large ferret trachea section. Only unspecific background staining is detectable for the polyclonal serum (#1, green) and the monoclonal antibody mix (#2, magenta). No signal overlap of both antibody signals was observed. Cyan/grayscale = autofluorescence. Edge length of grid squares = 800 µm. Total magnification = 2x.

**Table S1: List of reagents and antibodies used for immunostaining and optical clearing of tissue from SARS-CoV-2-infected ferrets.**

| **reagent** | **source** | **PO number** | **additional information** |
| --- | --- | --- | --- |
| DMSO | Carl Roth | 4720 | dimethyl sulfoxide |
| ethanol | Carl Roth | 9065 | dehydrating agent |
| ethyl cinnamate | Alfa Aesar | A12906 | clearing agent |
| Formical-2000™ | Statlab | 1314 | decalcifier |
| glycine | Carl Roth | 3908 | autofluorescence quencher |
| heparin sodium salt | Carl Roth | 7692 | reduction of background |
| hydrogen peroxide | Carl Roth | 8070 | bleaching agent |
| n-hexane | Alfa Aesar | 43263 | delipidating agent |
| normal donkey serum | Bio-Rad | C06SBZ | blocking agent |
| Triton X-100 | Carl Roth | 3051 | detergent |
| Tween-20 | AppliChem | A4974 | detergent |
| **antibody** | **source** | **PO number** | **additional information** |
| Alexa Fluor 488-labelled donkey anti-rabbit secondary antibody | Invitrogen | Cat#A-21206; RRID:AB_2535792 | dilution: 1:500 |
| Alexa Fluor 568-labelled donkey anti-mouse secondary antibody | Invitrogen | Cat#A10037; RRID:AB_2534013 | dilution: 1:500 |
| Alexa Fluor 568-labelled donkey anti-rabbit secondary antibody | Invitrogen | Cat# A10042; RRID:AB_2534017 | dilution: 1:500 |
| Alexa Fluor 647-labelled donkey anti-mouse secondary antibody | Invitrogen | Cat# A-31571; RRID:AB_162542 | dilution: 1:500 |
| mouse anti-SARS nucleocapsid antibody monoclonal (clone: 4F3C4) | Sven Reiche (FLI, Germany) | RRID:AB_2833162 | Bussmann et al., 2006; dilution 1:5 |
| mouse anti-SARS nucleocapsid monoclonal antibody (clone: 4E10A3A1) | Sven Reiche (FLI, Germany) | RRID:AB_2833160 | Bussmann et al., 2006; dilution 1:5 |
| rabbit anti-SARS nucleocapsid polyclonal antibody | Novus Biologicals | Cat#NB100-56576; RRID:AB_838838 | dilution: 1:250 |
